# Kidney-Protector Lipidic Cilastatin Derivatives as Structure-Directing Agents for the Synthesis of Mesoporous Silica Nanoparticles for Drug Delivery

**DOI:** 10.3390/ijms22157968

**Published:** 2021-07-26

**Authors:** Samuel Martinez-Erro, Francisco Navas, Eva Romaní-Cubells, Paloma Fernández-García, Victoria Morales, Raul Sanz, Rafael A. García-Muñoz

**Affiliations:** Department of Chemical and Environmental Technology, Rey Juan Carlos University (URJC), C/Tulipán s/n, Móstoles, 28933 Madrid, Spain; samuel.martinez@urjc.es (S.M.-E.); francisco.navas@urjc.es (F.N.); eva.romani.cubells@urjc.es (E.R.-C.); paloma.fgarcia@urjc.es (P.F.-G.); victoria.morales@urjc.es (V.M.); raul.sanz@urjc.es (R.S.)

**Keywords:** mesoporous silica nanoparticles (MSNs), drug-structure-directing agent (DSDA), cilastatin, drug delivery systems, sustained and controlled release

## Abstract

Mesoporous silica nanomaterials have emerged as promising vehicles in controlled drug delivery systems due to their ability to selectively transport, protect, and release pharmaceuticals in a controlled and sustained manner. One drawback of these drug delivery systems is their preparation procedure that usually requires several steps including the removal of the structure-directing agent (surfactant) and the later loading of the drug into the porous structure. Herein, we describe the preparation of mesoporous silica nanoparticles, as drug delivery systems from structure-directing agents based on the kidney-protector drug cilastatin in a simple, fast, and one-step process. The concept of drug-structure-directing agent (DSDA) allows the use of lipidic derivatives of cilastatin to direct the successful formation of mesoporous silica nanoparticles (MSNs). The inherent pharmacological activity of the surfactant DSDA cilastatin-based template permits that the MSNs can be directly employed as drug delivery nanocarriers, without the need of extra steps. MSNs thus synthesized have shown good sphericity and remarkable textural properties. The size of the nanoparticles can be adjusted by simply selecting the stirring speed, time, and aging temperature during the synthesis procedure. Moreover, the release experiments performed on these materials afforded a slow and sustained drug release over several days, which illustrates the MSNs potential utility as drug delivery system for the cilastatin cargo kidney protector. While most nanotechnology strategies focused on combating the different illnesses this methodology emphasizes on reducing the kidney toxicity associated to cancer chemotherapy.

## 1. Introduction

Mesoporous silica nanoparticles (MSNs) are materials comprising silica that bear pores with sizes in the range between 2 and 50 nm in their structure [1,2]. Over the past decades, MSNs have attracted much attention of the scientific community as they have found numerous applications in different fields of research. As some examples, MSNs have been widely applied in catalysis [3,4,5,6], as nanomaterials for energy storage and conversion [7,8], for processes of gas separation and water purification [9,10], and in biomedicine as nanomaterials for bioimaging, biosensing, or drug delivery [11,12,13,14,15,16,17].

The versatility and importance of MSNs is explained by the distinctive properties of these kinds of materials: high surface area and pore volume, high thermal stability, easy external surface functionalization, large and tunable pore sizes, and good biocompatibility [18,19]. These last two features are essential in the use of the MSNs as successful vehicles in drug delivery. Importantly, silica nanoparticles have shown no significant toxicity in numerous in vitro studies at concentrations around 100 μg mL^−1^ [20,21]. Other drug nanocarriers that have shown excellent biocompatibility are liposomes, niosomes, or iron nanoparticles [22,23,24]. The main difference is the existence in the MSNs of an extensive porous framework. This existence of mesoporous channels allows the accommodation of a wide variety of drugs and biological macromolecules (DNA, RNA, and proteins) and their subsequent diffusion and slow release [25,26,27,28,29].

The applications of MSNs in drug delivery are based on the encapsulation of a specific active or pro-active pharmaceutical ingredient within the structure of the MSNs for its subsequent release in the organism. This approach offers several advantages as opposed to the normal drug administration in single or in several doses [30,31]. First, it allows the controlled and sustained release of the drug in the body so that the therapeutic window can be extended over more time. At the same time, it improves the solubility and uptake of highly lipophilic drugs [32]. Moreover, the encapsulation of the drug inside the MSNs protects it as it avoids the premature metabolism of the drug [29,30,31,32,33].

The textural properties of the MSNs are extremely important for their applications in nanomedicine. The size, shape, pore size, and surface area of the materials have a huge influence on the behavior of the nanoparticles in the biological media [34,35]. The synthesis of the MSNs where all the features of the materials are set is, thus, of vital importance for their employment as drug delivery systems.

The synthesis of MSNs usually involves the use of templates (surfactants) which are able to self-assemble into micellar structures when the concentration reaches a certain concentration (critical micelle concentration, CMC). Quaternary ammonium salts under basic conditions or triblock copolymers under acidic conditions are typical examples of classic surfactants that have been used to effectively synthesize MSNs [17,29]. The silicon source (commonly tetraethoxysilane: TEOS or sodium silicate) is then added and the hydrolysis takes place around the supramolecular structures of the template micelles. As a result, a structured composite of silica around surfactant molecules is formed. In the final step of the synthesis, the organic templates are removed by calcination or solvent extraction to yield a mesoporous silica material with empty and accessible pores.

For their applications in drug delivery, an extra step is required later when the desired active pharmaceutical ingredient has to be adsorbed or loaded into the mesoporous structure. There are several methods that have been employed for the loading of a wide array of drugs into MSNs, the impregnation method being one of the most popular methods. It consists in the stirring of the suspension formed by the drug diluted in a solvent, and the MSNs for a certain amount of time. Then, the solvent is usually removed by evaporation [36,37].

In 2016, our group described a new concept on the synthesis of MSNs for their use in drug delivery; the synthesis of the drug-structure-directing agent (DSDA) [38,39]. During the synthesis of MSN, instead of using a conventional organic template that must be removed to load the drug into the pore structure, we proposed the employment of surfactants with inherent pharmacological activity. The same molecule that directs the structure of the mesoporous material is also responsible for the desired pharmaceutical activity in the body. In this manner, the synthesis of the MSNs as drug delivery systems is shortened, easier and, overall, more efficient.

**Figure 1:**
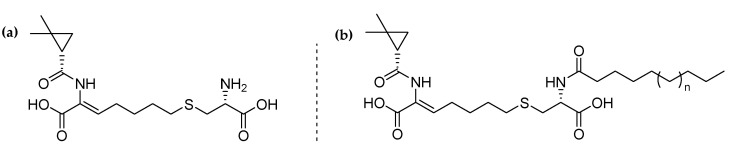
(**a**) Cilastatin and (**b**) drug-structure-directing agents from cilastatin.

Cilastatin (Figure 1a) is an inhibitor of dehydropeptidase DHP-I, an enzyme that is present in the kidney. Originally, this drug was developed to be administered in combination with the antibiotic imipenem as it can inhibit the metabolism and the tubular injury produced by imipenem [40]. Recent studies have also shown that cilastatin is able to reduce the renal impairment caused by several drugs used in cancer chemotherapy, such as cisplatin, vancomycin, and colistin, both in vitro and in vivo [41]. Cilastatin acts as a protector of the proximal tubular epithelial cells (PTECs) that suffer from apoptosis induced by the nephrotoxic drugs. Importantly, cilastatin does not affect the antibiotic or anticancer activity of the other drugs, but it reduces their inherent toxicity so that a higher dose of the medication can be administered without risk of nephrotoxicity [42,43]. Since in many patients with cancer the dosage of the drug must be diminished due to its renal toxicity, cilastatin would enhance the prognosis of cancer patients [44].

In this context, we envisioned the synthesis of mesoporous silica nanoparticles (MSNs) using lipidic derivatives of the drug cilastatin as structure-directing agents and as molecules with inherent pharmacological activity (Figure 1b). This method enables a simple, fast, and efficient protocol for the synthesis of MSNs, since it is not necessary to remove the surfactant of the synthesis and to load the drug, and therefore these MSNs could be directly employed as a drug delivery system of cilastatin without any extra steps. The MSNs materials exhibit substantially long and continuous cilastatin release times of up to 7 days. This investigation provides new opportunities for designing mesoporous silica nanocarriers as potentially intrinsic nanomedicines for different treatments and illnesses, mainly in patients with cancer since after intravenous injection, the drug would be biodistributed in the kidneys and therefore alleviating the nephrotoxicity of chemotherapy.

## 2. Materials and Methods

### 2.1. Chemicals

Cilastatin (95%) was purchased from Ambeed, Inc. (Arlington Hts, IL, USA) Oleoyl chloride (≥89%), decanoyl chloride (98%), tetrahydrofuran (THF, ≥99.0%), (3-aminopropyl)triethoxysilane (APTES, 98%), (3-aminopropyl)trimethoxysilane (APTMS, 97%), tetraethyl orthosilicate (TEOS, 98%), and hydrochloric acid (35% *w*/*w*) were obtained from Sigma-Aldrich (Madrid, Spain).

### 2.2. Synthesis of Anionic DSDAs from Cilastatin

The anionic DSDAs from cilastatin were obtained via amidation between the free α-amino group of cilastatin and decanoyl or oleoyl chloride (C_10_ or C_18_, respectively). The reaction was performed as published in our previous publication [38], with minor modifications. Cilastatin (1 g, 2.8 mmol, 1 equiv.) was dissolved in THF (10 mL) and the mixture was stirred at room temperature until the compound was completely dissolved. In another flask, a solution of NaOH (390 mg, 9.76 mmol, 3.5 mmol) in 10 mL of H_2_O was prepared. When this solution was cooled down, it was slowly added dropwise to the other mixture containing the cilastatin in THF with moderate stirring in an ice bath. After the addition, the corresponding carbonyl chloride (decanoyl or oleoyl chloride; 2.8 mmol, 1 equiv.) in THF (5 mL) was added dropwise over a period of 10 min. After that, the reaction was stirred overnight and let to reach room temperature. Then, water and hydrochloric acid were added until the mixture was brought to pH 2–3. The aqueous layer was then extracted with EtOAc (3 × 50 mL) and the combined organic layer were washed with H_2_O and brine (2 × 50 mL). The solvent was reduced under pressure to yield the corresponding lipidic derivative of cilastatin that was characterized by ^1^H and ^13^C NMR.

### 2.3. Synthesis of Mesoporous Silica Nanoparticles with DSDAs from Cilastatin (Cilastatin@MSNs)

In the optimal synthetic conditions, the MSNs synthesized using anionic DSDAs of cilastatin were obtained as followed. About 0.35 mmol of the anionic surfactant (cilastatin-C10 or cilastatin-C18) was emulsified in milli-Q water (15 mL) with stirring at 500 rpm at 100 °C overnight to obtain a concentration of 1.5 wt%. The solution was then cooled to 60 °C; (3-aminopropyl)triethoxysilane (137 μL, 0.585 mmol, 1.65 equiv.) was added and the resulting mixture was stirred at 500 rpm for 3 min. After that, tetraethyl orthosilicate (920 μL, 4.16 mmol, 11.8 equiv.) was added and the solution was stirred for 10 min at the same temperature and stirring rate. The stirrer was then removed from the solution and the mixture was allowed to stand at 60 °C overnight and finally at 100 °C for 1 day. The molar ratios of all components in the final mixture are 1.0/1.65/11.8/2360 of DSDA/APTES/TEOS/H_2_O. After the process, the precipitate was collected by filtration, washed with H_2_O, and dried under vacuum.

The reaction conditions were modified in some cases to study the effect of different factors on the textural properties of the nanoparticles. Increasing the ratio DSDA/APTES was studied as well as using APTMS instead of APTES. The effect of the stirring rate was also evaluated. The determination of the content of DSDA in the materials was determined by elemental analysis using the proportions of sulfur in the sample.

After many attempts in the synthesis of these kind of nanoparticles carrying the anionic DSDAs of cilastatin, optimal synthetic conditions were reached, with a high degree of reproducibility and repeatability.

### 2.4. Characterization

^1^H NMR and ^13^C NMR measurements were performed on a Varian Infinity 400 MHz spectrometer fitted with a 9.4 T magnetic field (URJC, Móstoles, Spain). Chemical shifts (δ) are shown in ppm and they were externally referenced to tetramethylsilane. Mass measurements were performed on an ultra-high-performance liquid chromatography-tandem mass spectrometry (UHPLC-HESI-MS/MS) using VIP heated electrospray ionization interface (Bruker UHPLC/MSMS EVOQ™ ELITE) with a triple-quadrupole detector (URJC, Móstoles, Spain).

The characterization of the morphology of the samples was performed with transmission electron microscopy (TEM) (National Centre of Electronic Microscopy, Madrid, Spain). The images of the MSNs were recorded using a JEOL JEM 2100 microscope operating at 200 kV and with a resolution of 0.25 nm. Previously, the samples were dispersed in ethanol and deposited on a carbon-coated copper grid. We also used a JEOL JEM 1400 Flash microscope at 120 kV for the same purpose (Microscopy Service of Universitat Politècnica de València, València, Spain). The samples were dispersed (0.04 mg mL^−1^) in ultrapure water (18.2 MΩ cm) and transferred to cupper square mesh grids.

The textural properties were obtained from the N_2_ adsorption–desorption isotherms at −196 °C using a Micromeritics TriStar 3000 instrument (URJC, Móstoles, Spain). Prior to the measurement, the samples were first calcinated at 550 °C during 5 h and then outgassed at 100 °C for 24 h with a N_2_ flux (URJC, Móstoles, Spain). Cylindrical pore geometry was assumed for the calculation of the mesopore size distribution (PSD) using the NLDFT model.

FTIR analyses were collected, using the KBr buffer technique, on a Mattson Infinity series apparatus in the wavelength range from 4000 to 400 cm^−1^ with a step size of 2 cm^−1^ and collecting 64 scans for each analysis (URJC, Móstoles, Spain). XRD patterns were recorded from the calcinated samples on a Philips X’PERT MPD powder diffractometer equipped with CuKα radiation (URJC, Móstoles, Spain).

Thermogravimetric analyses were performed under air atmosphere with a Star system Mettler Thermobalance in the temperature range from 40 to 800 °C at 5 °C min^−1^ (URJC, Móstoles, Spain). The elemental analyses of the samples were done with a CHNS-O analyzer Flash 2000 Thermo Scientific apparatus (URJC, Móstoles, Spain). A NanoPlus DLS Zeta potential from Micromeritics was used for obtaining the zeta potential values of the particle suspensions (URJC, Móstoles, Spain). The samples were suspended in phosphate buffered saline (PBS, pH = 7.4) with 1 mg mL^−1^ concentration. The buffer was prepared by combining 8 g of NaCl, 200 mg of KCl, 1.44 g of Na_2_HPO_4_, 245 mg of KH_2_PO_4_ in 800 mL of distilled water. After adjusting the pH to 7.4 the solution was filled until reaching 1 L.

### 2.5. Drug Delivery Studies

The release studies of cilastatin were performed in phosphate buffered saline (PBS, pH = 7.4). The experiment was conducted as follows: 50 mg of dry MSNs were immersed in 50 mL of PBS solution at 37 °C with stirring and 1 mL of the solution was removed at certain times for the monitorization. After centrifugation to remove the nanoparticles, the concentration of the drug in solution was calculated using a UV-spectrometer (JASCO V-630) at a maximum of 206 nm.

## 3. Results and Discussion

### 3.1. Synthesis and Characterization of the Anionic DSDAs of Cilastatin

The first goal of this work was to obtain derivatives of the drug cilastatin that may be employed as drug-structure-directing agents (DSDAs). For this purpose, the molecule must have an amphiphilic character with a hydrophilic and a hydrophobic part. In this case, cilastatin bears two carboxylic groups, so the non-polar carbon chain must be added to the molecule. The introduction of the fatty acid chain was performed through amidation of the α-amino group of the drug and the carbonyl group of decanoyl or oleoyl chloride. These two fatty acid derivatives were selected in order to have an insight of a short and a long lipidic chain.

The formation of the desired amide bond was ubiquitously confirmed using ^1^H and ^13^C NMR spectroscopy. The new signal of the proton of the –N*H* group of the amide can be detected as a doublet at 8.13 ppm and at 8.08 ppm in the spectrum of cilastatin-C10 and cilastatin-C18, respectively (See supplementary information, Figures S1 and S4). The deshielding of the signal of proton bound to the α-carbon of the amino acid (4.36 ppm in both spectra) is also indicative of the correct formation of the amide bond. Importantly, in the ^13^C NMR spectrum of both DSDAs, the signal of the newly formed amide carbon is also present at 172.9 ppm for the compound with a C10 chain and at 172.3 ppm for the one with C18 (SI, Figures S1 and S4). The organic compounds were also characterized using FTIR measurements (SI, Figures S2 and S5), where the stretching vibrations bands of the multiple C-H bonds of the fatty acid chains can be observed between 3000 and 4000 cm^−1^. Finally, mass spectrometry in the positive method was employed to correctly detect the mass peaks of both DSDAs (Figures S3 and S6). Both mass spectra show the signals corresponding to the exact mass of each DSDA. Besides, these surfactants have two free carboxylic acids in their structure (*vide supra*, Figure 1). This fact was confirmed with Z potential measurements that showed values of −6.1 mV and −12.8 mV for cilastatin-C10 and cilastatin C-18 respectively.

### 3.2. Synthesis and Characterization of Mesoporous Silica Nanoparticles with DSDAs of Cilastatin (cilastatin@MSNs)

As previously mentioned, the textural properties of MSNs are set during the synthetic procedure of the nanoparticles, so it is a particularly critical step in materials designed to be employed as nanoplatforms in drug delivery. In particular, the size and shape of the whole nanoparticles and the size of the pores are two of the most relevant characteristics. It is well stablished that the size and shape of MSNs have an important impact on their interaction inside of living organisms [45,46]. In general, the optimal nanoparticles must be spherical and porous so they can be able to have a large store capacity and controlled delivery [47]. With these objectives in mind, we started to evaluate different reaction conditions for the synthesis of MSNs using DSDAs of cilastatin-C10 and C18. These surfactants have anionic nature because of the two carboxylic acids of cilastatin. In consequence, a co-structure directing agent (CSDA) is required for the adequate formation of the micelles, as previously described [38,48]. In this work, we chose to start using 3-aminopropyltrimethoxy silane (APTMS) as the source of CSDA. Since cilastatin has two free carboxylic acids, we add double the amount of the co-structure directing agent. In this way, we foresaw that all negatively charged groups of cilastatin would interact with the positively charged groups of APTMS to correctly form the micelles. Then, tetraethoxysilane (TEOS) will be employed as the source of silica to condense around the micelles and form the nanoparticles.

**Figure 2:**
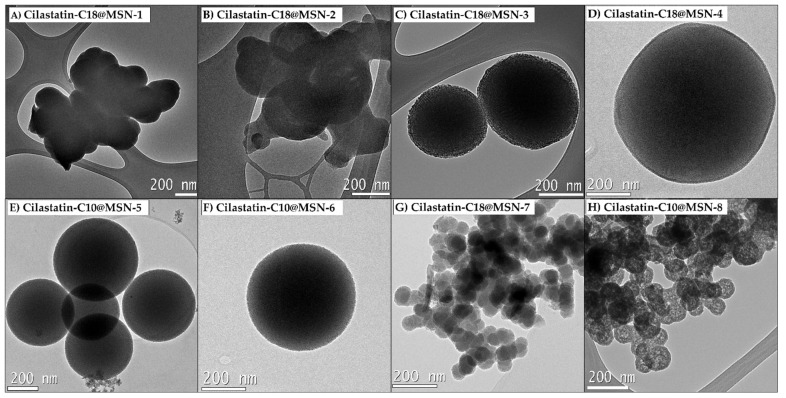
TEM images of the materials synthesized according to the reaction conditions in Table 1. (**A**) Cilastatin-C18@MSN-1; (**B**) Cilastatin-C18@MSN-2; (**C**) Cilastatin-C18@MSN-3; (**D**) Cilastatin-C18@MSN-4; (**E**) Cilastatin-C10@MSN-5; (**F**) Cilastatin-C10@MSN-6; (**G**) Cilastatin-C10@MSN-7; (**H**) Cilastatin-C10@MSN-8.

In the first attempt of the synthesis and based on our investigations [38], we performed the reaction with 1 equiv. of the DSDA of cilastatin-C18 in milli-Q water with a concentration of 1.5 wt%. As mentioned above, we use double amount of APTMS than what was previously reported (3.3 equiv.) and 11.8 equiv. of TEOS. After the addition of APTMS, the reaction was stirred for 5 min. at 500 rpm before adding the TEOS. About 10 min later, the stirrer was removed, and the reaction was let to stand at 60 °C overnight and at 100 °C for 1 day (Table 1, entry 1). The transmission electron microscopy (TEM) images of the synthesized materials were used to check the morphology and size of the nanoparticles cilastatin-C18@MSNs-1 (Figure 2).

The MSNs synthesized with the reaction conditions depicted in entry 1 yielded an amorphous material without spherical morphology and an extensive aggregation was observed (Figure 2A). The N_2_ isotherm of the calcined material showed a BET surface area of 606 m^2^ g^−1^ and a pore volume of 0.42 cm^3^ g^−1^ (Table 2, entry 1). We believed that the extensive aggregation observed in the micrographs of TEM could be due to the excessive amount of the co-structure directing agent (APTMS), so we tested the experiment with half the equivalents of CSDA (Table 1, entry 2). The nanoparticles obtained in this manner showed less agglomeration and more sphericity than the previous material, but they were not discrete with smooth edges. The textural properties of MSNs were also determined yielding a BET surface area of 375 m^2^ g^−1^ (Table 2, entry 2). We envisioned that the morphology of the nanoparticles was negatively being affected by the high polymerization rate of APTMS. For this reason, we continued our optimization process by changing the co-structure directing agent to 3-aminopropyltriethoxysilane APTES, which is described to have a slower polymerization rate (Table 1, entry 3) [49].

Fortunately, we managed to get highly dispersed spherical nanoparticles with a small size of around 300–400 nm (Figure 2C). We hypothesized that APTMS, apart from interacting with the negatively groups of cilastatin and forming the micelles, started to polymerize very early causing agglomeration of the particles (Figure 2A,B). On the other hand, the slower polymerization rate of APTES, allowed the correct formation of dispersed micelles before the addition of TEOS and the subsequent polymerization.

**Figure 3:**
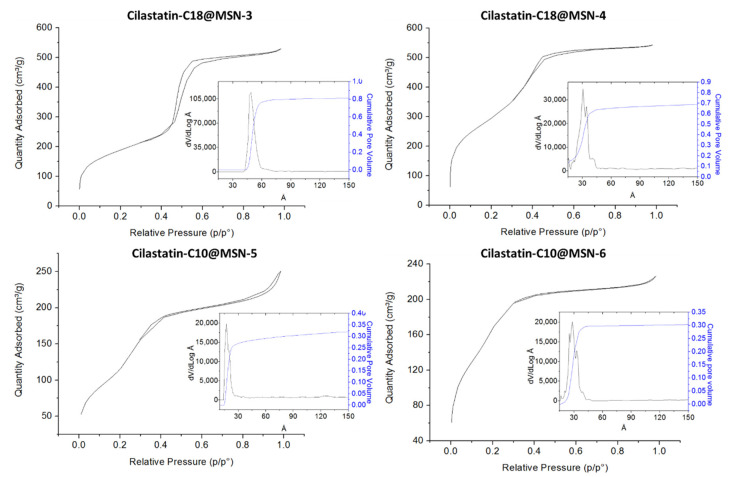
N_2_ adsorption–desorption isotherms and pore size distributions (PSD) based on a NLDFT model of Cilastatin-C18@MSN-3, Cilastatin-C18@MSN-4, Cilastatin-C18@MSN-5, and Cilastatin-C18@MSN-6.

The textural properties of these MSNs were obtained from the isothermal N_2_ adsorption–desorption experiments of the calcinated material (Figure 3). Cilastatin-C18@MSN-3 showed a remarkable BET surface area of 685 m^2^ g^−1^ (Table 2, entry 3). The total pore volume was also quite large (0.80 cm^3^ g^−1^) and the pore size distribution showed a maximum at 4.9 nm.

The effect of the reaction conditions on the MSNs was next studied (Table 1, entry 4). The reaction was performed with the thermal treating at 60 °C overnight but without the aging at 100 °C for an extra day (Cilastatin-C18@MSN-4). While the obtained MSNs were very similar both in shape and size (Figure 2D), cilastatin-C18@MSN-4 showed a significantly larger BET surface area of 1073 m^2^ g^−1^ (Table 2, entry 4). The total pore volume was 0.83 cm^3^ g^−1^ and the pore size distribution revealed a mesopore distribution in between 3.0 and 4.5 nm with a maximum at 4.1 nm. We performed the same experiments with the other DSDA under investigation, cilastatin-C10. Using the same reaction conditions, we obtained cilastatin-C10@MSN-5 and cilastatin-C10@MSN-6, the only difference being that in the latter one we omitted the thermal aging treatment of the nanoparticles (Table 1, entries 5 and 6). In this case, the MSNs also presented spherical morphology but with sizes of around 500 nm. As occurred with cilastatin-C18, the morphology of the particles was not affected by the thermal aging (Figure 2E,F). The BET surface area of cilastatin-C10@MSN-5 was determined to be 496 m^2^ g^−1^ and the pore volume was 0.35 cm^3^ g^−1^. The sample reveals mesopores with a maximum of 2.0 nm in the pore size distribution (Table 2, entry 5). The textural properties of the sample without the thermal aging also showed an increased BET surface area with a value of 632 m^2^ g^−1^ (Entry 6).

Encouraged by these results, we decided to continue our investigations with the aim of reducing the size of the nanoparticles. As previously mentioned, small nanoparticles are known to have better behavior in biological systems as they can be easily internalized by cells via endocytosis [50]. We hypothesized that increasing the stirring rate during the emulsion of the DSDA and during the reaction would reduce the size of the micelles and that, in turn, would result in smaller nanoparticles.

Effectively, when we tried the same conditions as cilastatin-C18@MSN-3 but with an increased stirring rate (Table 1, entry 7), we obtained spherical MSNs with very small particle sizes of around 50 nm (cilastatin-C18@MSN-7, Figure 2G). The effect of the increased stirring rate was also evaluated with the other DSDA, cilastatin-C10 (Table 1, entry 8). In this case, we were also able to decrease the particle size significantly to around 70 nm (Figure 2H).

**Figure 4:**
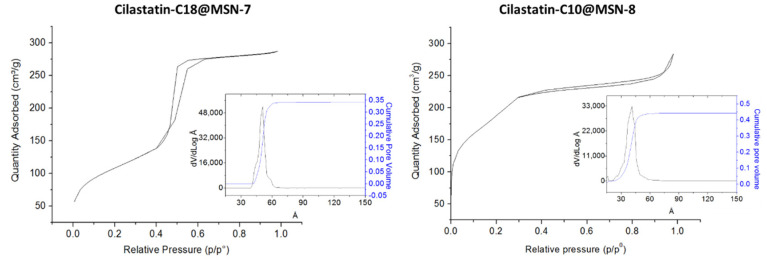
N_2_ adsorption–desorption isotherms and pore size distributions (PSD) based on a NLDFT model of Cilastatin-C18@MSN-7 and Cilastatin-C10@MSN-8.

The textural properties of these smaller MSNs were also determined (Table 2, entries 7–8 and Figure 4). Cilastatin-C18@MSN-7 provided a BET surface area of 383 m^2^ g^−1^ and a total pore volume of 0.35 cm^3^ g^−1^ (Table 2, entry 7). The pore size distribution was in between 4.5 nm and 5.5 with a maximum at 5.0 nm. The MSNs synthesized with the DSDA of cilastatin-C10 and an increased stirring rate afforded very promising textural properties. Cilastatin-C10@MSN-8 gave a BET surface area of 673 m^2^ g^−1^, a total pore volume of 0.47 cm^3^ g^−1^, and a mesoporous distribution with a maximum at 3.1 nm (Table 2, entry 8).

The synthesized cilastatin-C18@MSNs and cilastatin-C10@MSNs were also characterized using low angle X-ray diffraction and FTIR measurements (See supplementary information, Figures S7 and S8). The XRD patterns of the calcinated materials show a band at around 2 theta (2θ degrees) which may suggest a specific ordering of the pores in these cilastatin@MSNs. The materials show the characteristic IR bands related to MSNs: –Si–O–Si– bending bands (450–500 cm^−1^), –Si–O–Si– symmetric stretching bands (760–800 cm^−1^), and –Si–O–Si– asymmetric stretching bands (1050–1100 cm^−1^). Importantly, all cilastatin@MSNs have also IR bands in the region that corresponds to the stretching of C–H bonds (2850–3000 cm^−1^). This fact evidences the correct incorporation of the organic, pharmaceutically active DSDA into the structure of the MSNs. The thermogravimetric analysis of the materials synthesized also confirms the presence of the organic surfactant within the structure of the mesoporous silica. An organic content in the range of 28 and 41% was determined between the as-made material and the calcined sample (SI, Figure S9).

### 3.3. Release Experiments of Mesoporous Silica Nanoparticles with DSDAs of Cilastatin (cilastatin@MSNs)

The release studies were conducted at 37 °C in PBS in order to get a simulation of the biological media of the human body. Two different MSNs were selected for the conduction of the release experiments: cilastatin-C18@MSN-3 and cilastatin-C10@MSN-8. These materials were chosen because of they have a similar BET surface area of 685 and 673 m^2^ g^−1^, respectively. The goal was to study the difference of the release of the two DSDAs (C10 and C18) in different MSNs. After release from the DSDA and cleavage of the amide [38], the DSDA renders decanoic or oleic acid and cilastatin kidney-protector drug, separately, in equimolar amounts.

**Figure 5:**
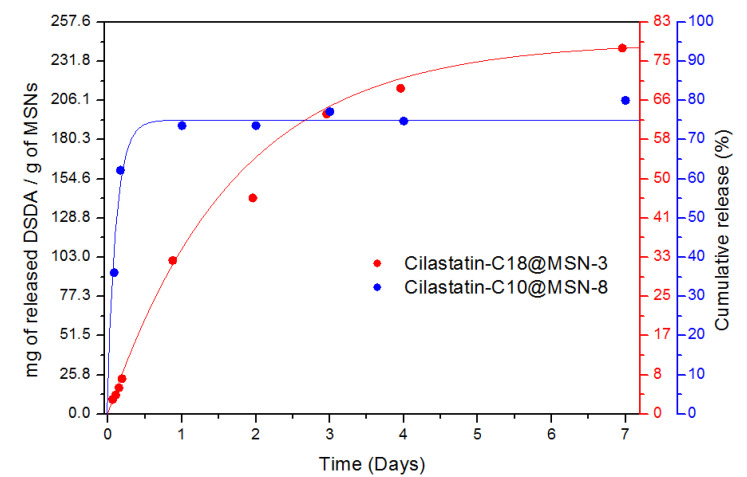
Release experiments of Cilastatin-C18@MSN-3 (red) and Cilastatin-C10@MSN-8 (blue).

The amount of DSDA released per gram of MSNs was plotted against time to construct the release profile for each DSDA (Figure 5). Both materials showed a slow and sustained release of the drug during several days. Cilastatin-C18@MSN-3 released 100 mg of cilastatin-C18 in 1 day and 200 mg in 4 days. These amounts correspond to percentages of around 32% of the maximum theoretical release after 1 day and 68% after 4 days. Cilastatin-C18@MSN-3 reaches a maximum release of around 78% after 7 days which is equivalent to around 240 mg of cilastatin-C18 per gram of MSN (Figure 5 in red). This material shows a slow and sustained release of the drug over a long period of time, which makes it an adequate system to be employed in drug delivery.

Next, cilastatin-C10@MSN-8 was set under the same delivery conditions in order to study the release of the DSDA in this material. In comparison, cilastatin-C10 was released from the nanoparticles in higher amount in shorter times than cilastatin-C18 (Figure 5 in blue). After only 4 h, 160 mg of DSDA were released to the media which corresponds to 62% of the maximum theoretical release. After 1 day, 190 mg of DSDA were released (73%) and that amount continued essentially constant after 1 week when the total released reached around 80%. Therefore, cilastatin-C18 and cilastatin-C10 species were observed to release from the mesoporous silica hosts at significantly different rates. The differences in the release behavior from these materials can be explained due the different solubilities and sizes of the two DSDAs and the different morphologies of the nanoparticles. The smaller size and higher hydrophilicity of cilastatin-C10 allows its easier diffusion from the cavities of the nanoparticles and its subsequent earlier release to the media. On the other hand, the lower hydrophobicity and higher size of DSDA cilastatin-C18 allows a slower and more continuous release overtime. The bigger particle size of cilastatin-C18@MSN-3 could also be responsible for the slower release. The molecules of DSDA located in the interior of the particle have to overcome more interactions with the matrix of the MSN to be released, which, in turn, result in a slower release of the drug. The data obtained in the release experiments have been fitted to two different drug-release models [22,24], the Higuchi and the Korsmeyer–Peppas model (Supporting Information, Table S1). The correlation value (R^2^) suggests that the Korsmeyer–Peppas model might be a better descriptor of the release of the DSDA in both cilastatin-C18@MSN-3 and cilastatin-C10@MSN-8.

Bearing in mind the previous results and considering the target of these nanoparticles is the renal tissue, upon administration either via oral or by intravenous injection, the nanoparticles and the released cilastatin DSDA from nanoparticles would reach the bloodstream. From here and due to their small size (<5 nm) [51], the lipidic derivatives of cilastatin would undergo renal clearance reaching in this manner the objective, allowing their accumulation in the kidneys, and thus imparting the desired therapeutic effect in the target tissue.

## 4. Conclusions

In summary, we have successfully synthesized lipidic derivatives of kidney-protector cilastatin to be employed as structure-directing agents for the synthesis of mesoporous silica nanoparticles (MSNs). The inherent pharmacological activity of the surfactant allows the straightforward synthesis of MSNs for their use in drug delivery without the need of any extra step. All nanomaterials synthesized showed good sphericity and excellent textural properties with BET surface areas of up to 1073 m^2^ g^−1^ and total pore volumes of up to 0.83 cm^3^ g^−1^. The size of the nanoparticles could be reduced by increasing the stirring rate during the synthesis to obtain materials with very small particle sizes of 50 to 70 nm.

Importantly, the release studies of these materials revealed a sustained and controlled release of the drug to the media, which proves their potential utility as drug delivery systems for cilastatin. The material obtained with the DSDA cilastatin-C18 showed a slower release profile with a 32% of DSDA released after 1 day and 64% after 4 days. The MSNs obtained with cilastatin-C10 revealed a faster release of the DSDA with a 64% after 4 h and 73% in 1 day. Since cilastatin has shown promising effects against nephrotoxic agents such as vancomycin or cisplatin, the obtention of these MSNs could allow a higher protective effect on the kidney during chemotherapy treatments. The release experiments obtained in this work, suggest that cilastatin@MSNs would generate a continuous release of the drug overtime allowing the protection of the kidney in long treatments with nephrotoxic drugs. While most nanotechnology strategies for cancer treatment are focused on cytotoxicity, this methodology emphasizes on reducing the kidney toxicity associated to cancer chemotherapy and thus relief the anticancer drugs adverse effects.

## Figures and Tables

**Table 1:** Synthesis conditions of mesoporous silica nanoparticles with DSDAs of cilastatin-C10 and C18 (cilastatin-C10@MSNs and cilastatin-C18@MSNs).

Entry	Nanomaterial	Equiv. of CSDA	Stirring Rate	Conditions ^1^
1	Cilastatin-C18@MSN-1	3.3 equiv. of APTMS	500 rpm	60 °C o.n. then 100 °C 1 day
2	Cilastatin-C18@MSN-2	1.65 equiv. of APTMS	500 rpm	60 °C o.n. then 100 °C 1 day
3	Cilastatin-C18@MSN-3	1.65 equiv. of APTES	500 rpm	60 °C o.n. then 100 °C 1 day
4	Cilastatin-C18@MSN-4	1.65 equiv. of APTES	500 rpm	60 °C o.n.
5	Cilastatin-C10@MSN-5	1.65 equiv. of APTES	500 rpm	60 °C o.n. then 100 °C 1 day
6	Cilastatin-C10@MSN-6	1.65 equiv. of APTES	500 rpm	60 °C o.n.
7	Cilastatin-C18@MSN-7	1.65 equiv. of APTES	800 rpm	60 °C o.n.
8	Cilastatin-C10@MSN-8	1.65 equiv. of APTES	800 rpm	60 °C o.n.

^1^ o.n. = overnight.

**Table 2:** Mesoporous silica nanoparticles textural properties of cilastatin-C10@MSNs and cilastatin-C18@MSNs.

Entry	Nanomaterial	BET (m^2^ g^−1^)	Vp [cm^3^ g^−1^]	PSD [nm] ^1^	D_N_ (nm) ^2^
1	Cilastatin-C18@MSN-1	606	0.42	4.5 (3.5–5.0)	n.d.
2	Cilastatin-C18@MSN-2	375	0.26	4.7 (2.5–5.5)	n.d.
3	Cilastatin-C18@MSN-3	685	0.80	4.9 (4.5–5.5)	373 ± 75
4	Cilastatin-C18@MSN-4	1073	0.83	4.1 (3.0–4.5)	744 ± 165
5	Cilastatin-C10@MSN-5	496	0.35	2.0 (1.5–2.5)	553 ± 149
6	Cilastatin-C10@MSN-6	632	0.32	2.8 (2.2–3.5)	406 ± 177
7	Cilastatin-C18@MSN-7	383	0.35	5.0 (4.5–5.5)	50 ± 20
8	Cilastatin-C10@MSN-8	673	0.47	3.1 (2.5–3.5)	69 ± 29

^1^ Pore size distribution (PSD) based on NLDFT model. Maximum of the distribution and PSD in brackets. ^2^ Determined from TEM micrographs. n.d. = not determined.

## Data Availability

The raw/processed data required to reproduce these findings cannot be shared at this time as the data also forms part of an ongoing study.

## Supplementary Materials

The following are available online at https://www.mdpi.com/article/10.3390/ijms22157968/s1.
