# Supplementary material for: Kidney-Protector Lipidic Cilastatin Derivatives as Structure-Directing Agents for the Synthesis of Mesoporous Silica Nanoparticles for Drug Delivery
Source: Int J Mol Sci. 2021 Jul 26;22(15):7968. doi: 10.3390/ijms22157968 (PMC8348040; doi:10.3390/ijms22157968)
Supplement: Supplementary file 1 [file ijms-22-07968-s001.zip › ijms-1312755-supplementary.pdf]

Kidney-Protector Lipidic Cilastatin Derivatives as Drug-Structure-Directing Agents for the Synthesis of MSNs Drug Delivery Systems

Supporting Information

Characterization of the anionic DSDAs of cilastatin

Cilastatin-C10

^{1}H NMR (DMSO-d_6): δ 12.41 (s, 2H); 9.11 (s, 1H); 8.13 (d, 1H, J = 7.2 Hz); 6.33 (t, 1H, J = 7.2 Hz); 4.36 (m, 1H); 2.87 (dd, 1H, J = 13.3, 4.5 Hz); 2.69 (dd, 1H, J = 13.3, 4.5 Hz); 2.50 (t, 2H, J = 7.9 Hz); 2.18-2.02 (m, 4H); 1.61 (m, 1H); 1.46 (m, 6H); 1.24 (s, 12H); 1.10 (m, 6H); 0.85 (t, 3H, J = 7.9 Hz); 0.69 (m, 1H). **^{13}C RMN (DMSO-d_6):** δ 175.3, 173.0, 170.3, 166.5, 135.8, 129.0, 52.6, 35.7, 33.6, 32.0, 29.6, 29.5, 29.4, 29.3, 29.2, 28.3, 27.5, 27.4, 25.9, 22.8, 21.7, 20.2, 19.2, 14.6. **ESI MS $[M+H]^+$** = 513.1.

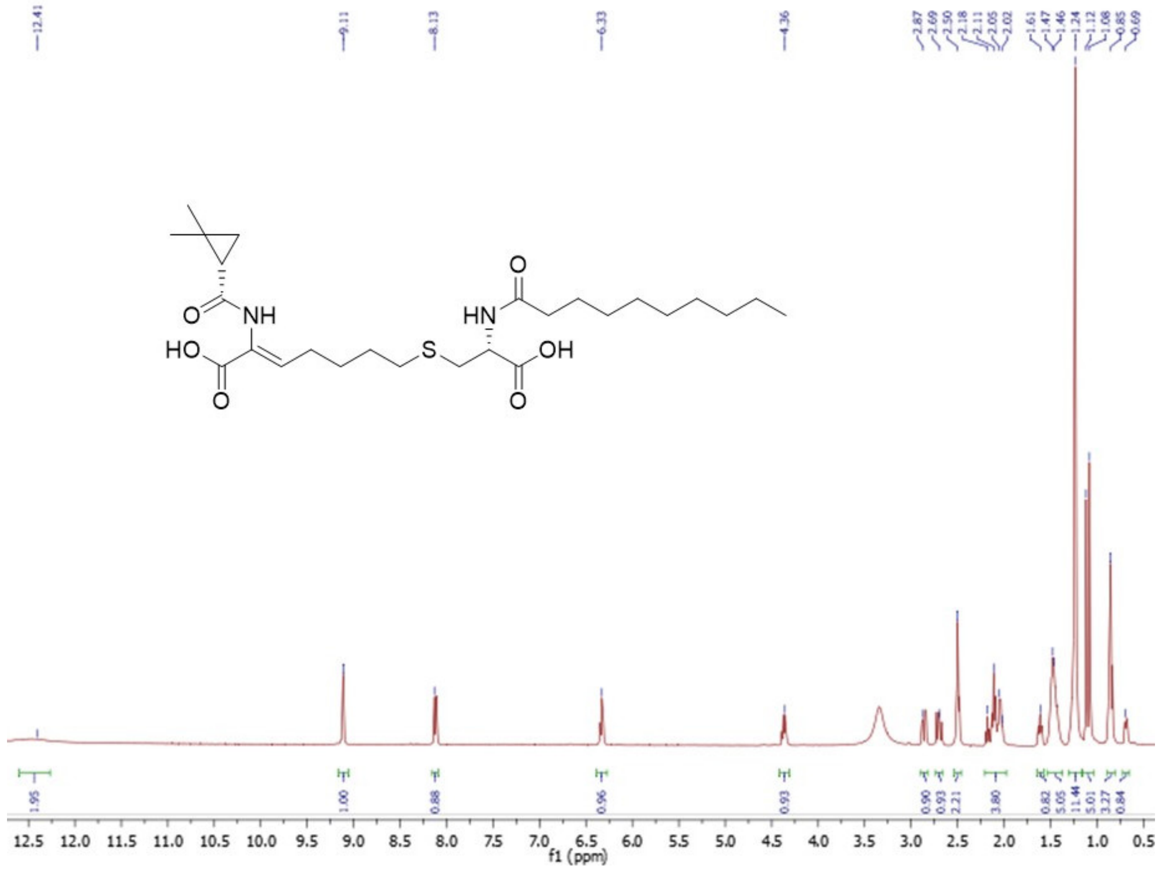

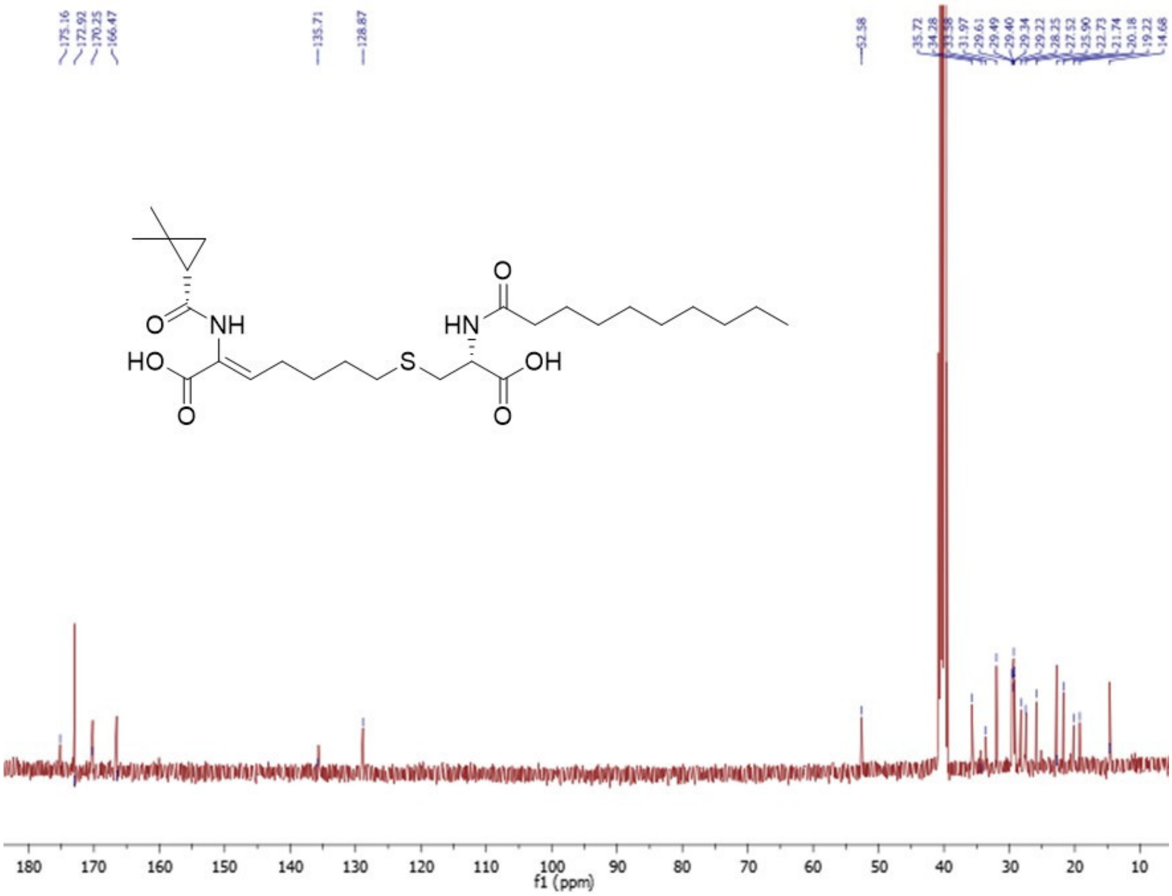

Figure S1. ^{1}H NMR and ^{13}C NMR spectra of the DSDA cilastatin-C10 in DMSO-d_6.

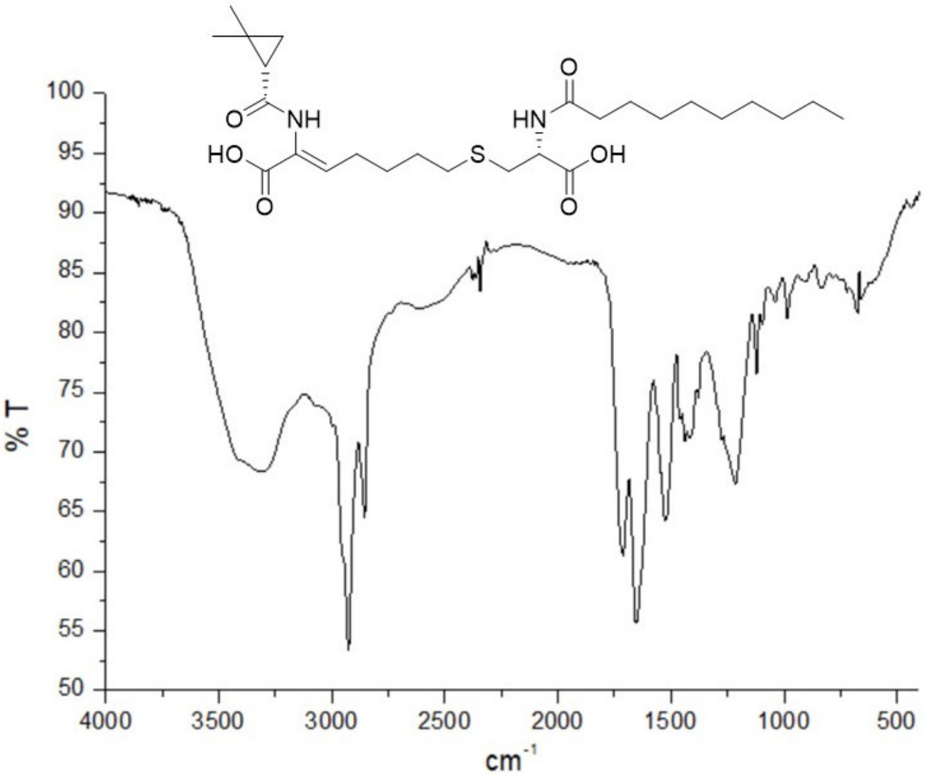

Figure S2. FTIR measurements of the DSDA cilasatin-C10.

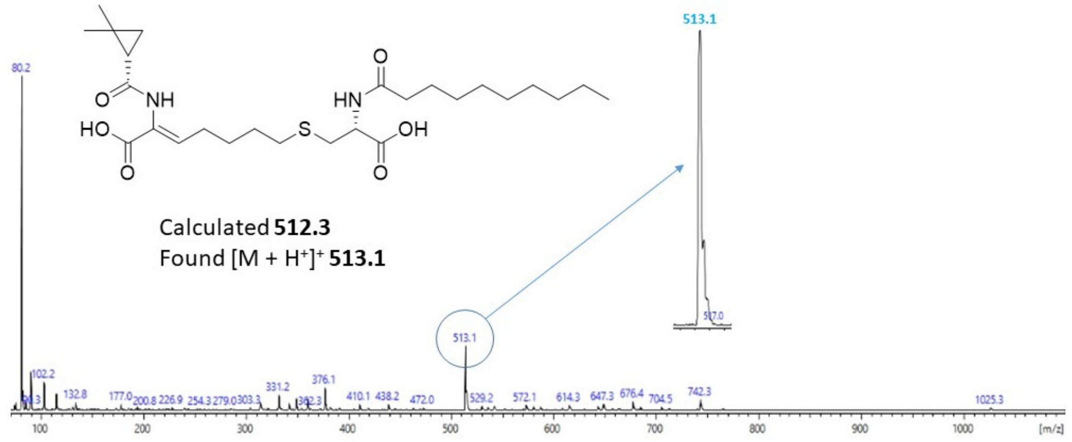

Figure S3. ESI-Mass spectrum of the DSDA cilastatin-C10.

Cilastatin-C18

^{1}H NMR (DMSO-d_6): δ 9.11 (s, 1H); 8.08 (d, 1H, J = 7.9 Hz); 6.33 (t, 1H, J = 7.2 Hz); 5.32 (m, 2H) ; 4.36 (m, 1H); 2.86 (dd, 1H, J = 13.3, 4.5Hz); 2.70 (dd, 1H, J = 13.3, 4.5Hz); 2.15 (t, 2H, J = 7.9 Hz); 2.09 (m, 2H); 2.01 (m, 2H) 1.97 (m, 6H); 1.60 (t, 2H, J = 7.9 Hz); 1.44 (d, 1H, J = 7.9 Hz); 1.24 (s, 24H); 1.06 (m, 6H); 0.83 (t, 3H, J = 7.9 Hz); 0.67 (m, 1H). **^{13}C RMN (DMSO-d_6):** δ 174.5, 172.3, 169.5, 165.9, 134.9, 129.6, 128.3, 52.0, 35.1, 33.7, 33.0, 31.3, 29.1, 28.8, 28.7, 28.6, 27.6, 26.6, 25.2, 22.1, 21.0, 19.9, 18.5, 13.9. **ESI MS [M+H]$^+$** = 623.4.

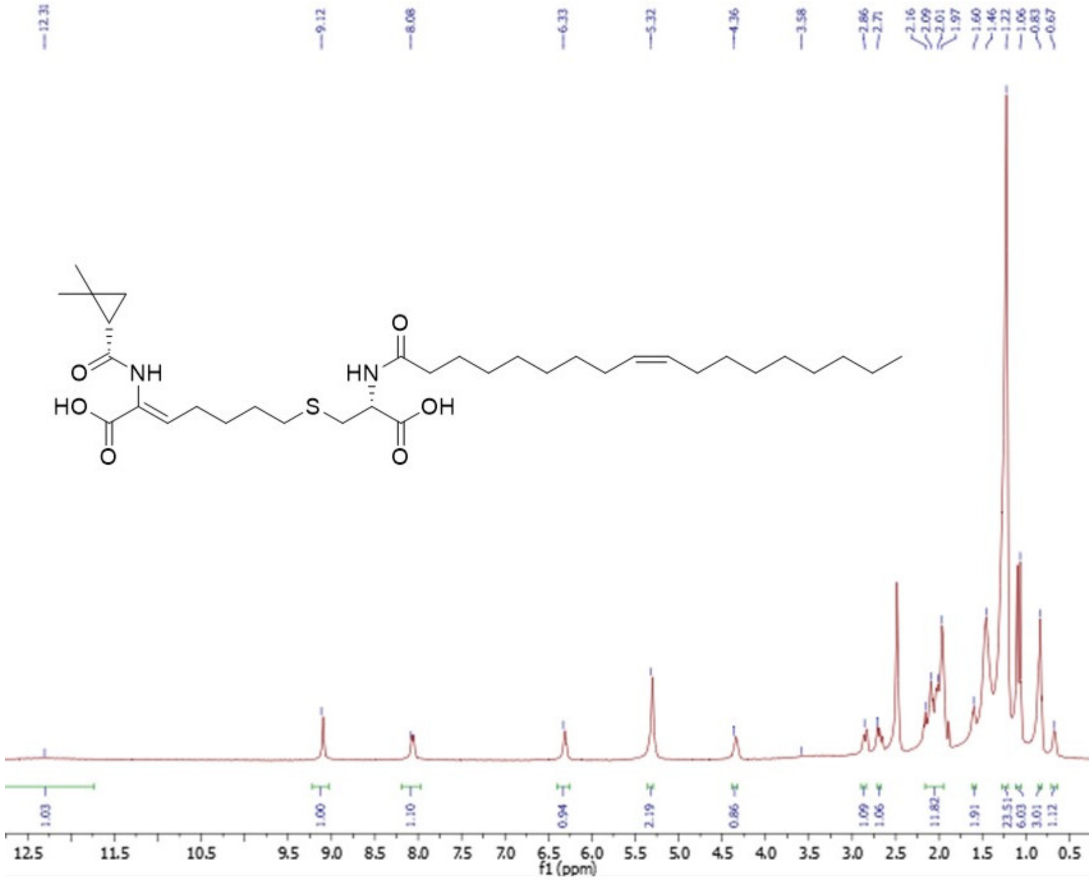

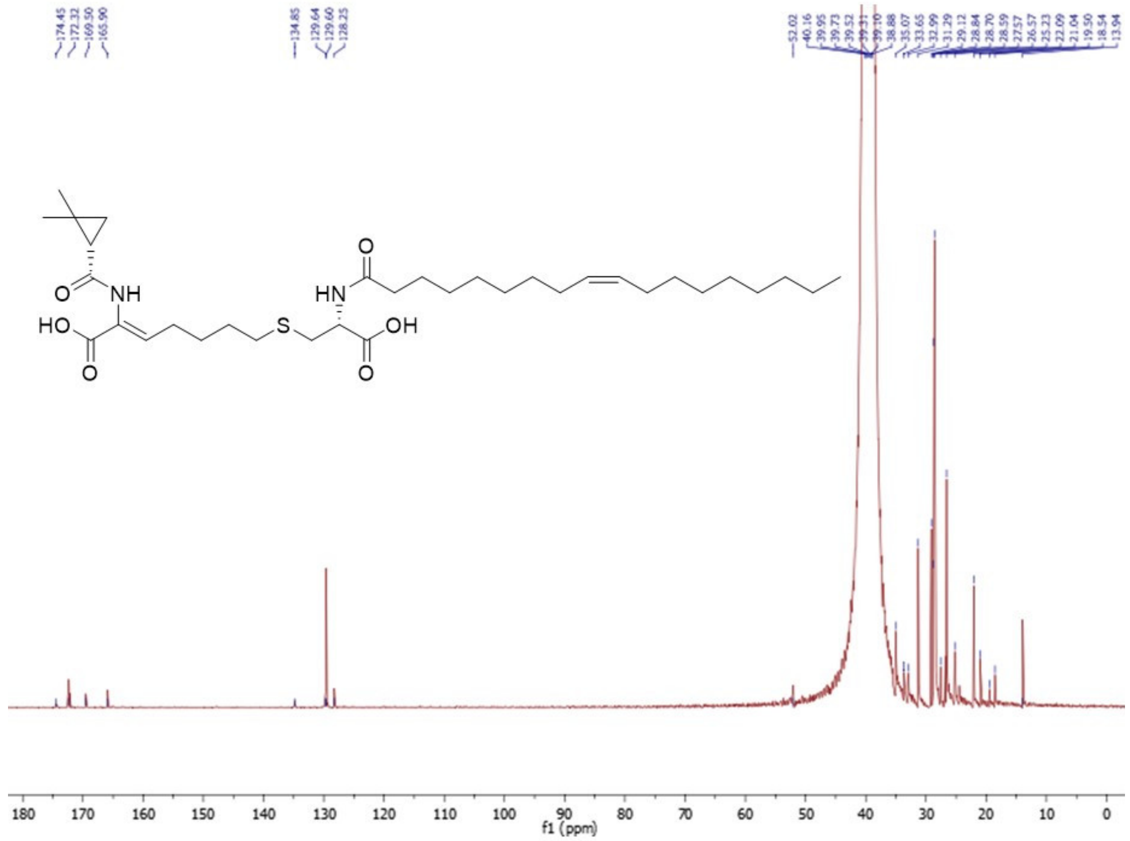

Figure S4. ^{1}H NMR and ^{13}C NMR spectra of the DSDA cilastatin-C18 in DMSO-*d*$_{6}$.

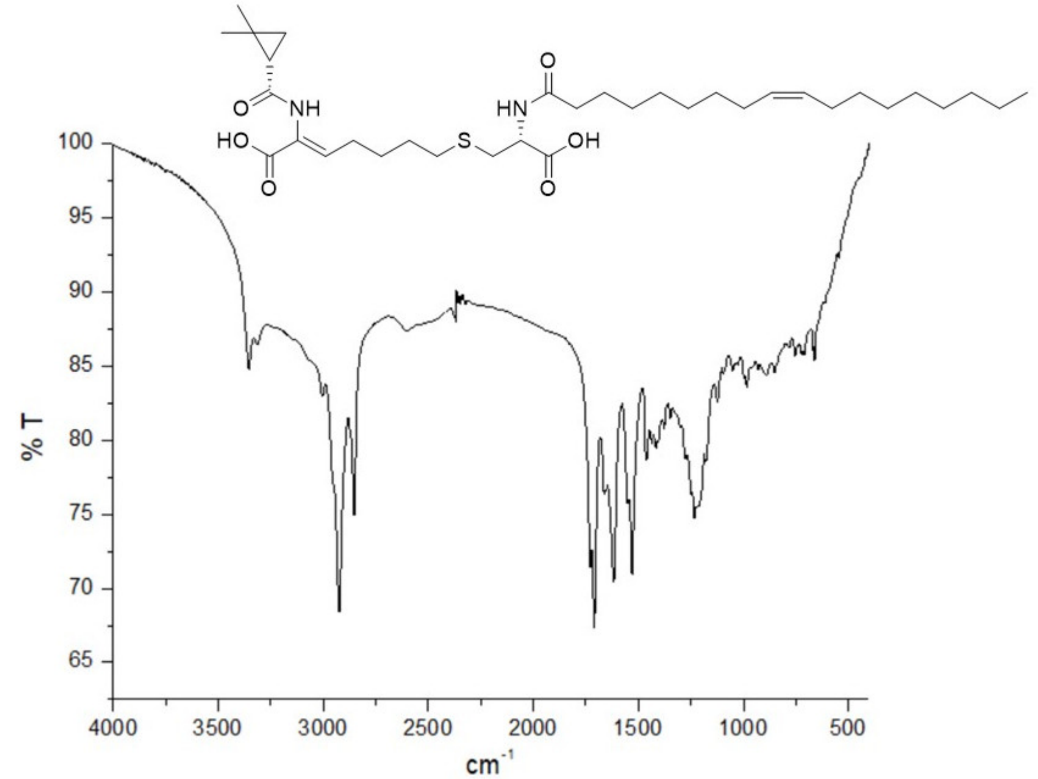

Figure S5. FTIR measurements of the DSDA cilasatin-C18.

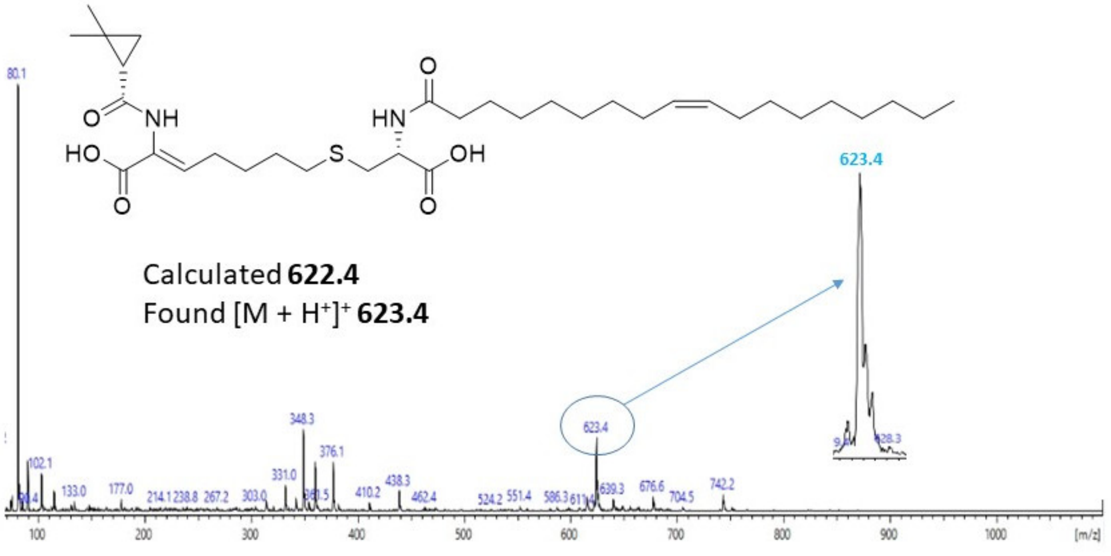

Figure S6. ESI-Mass spectrum of the DSDA cilastatin-C18.

Characterization of mesoporous silica nanoparticles with DSDAs of cilastatin (cilastatin@MSNs):

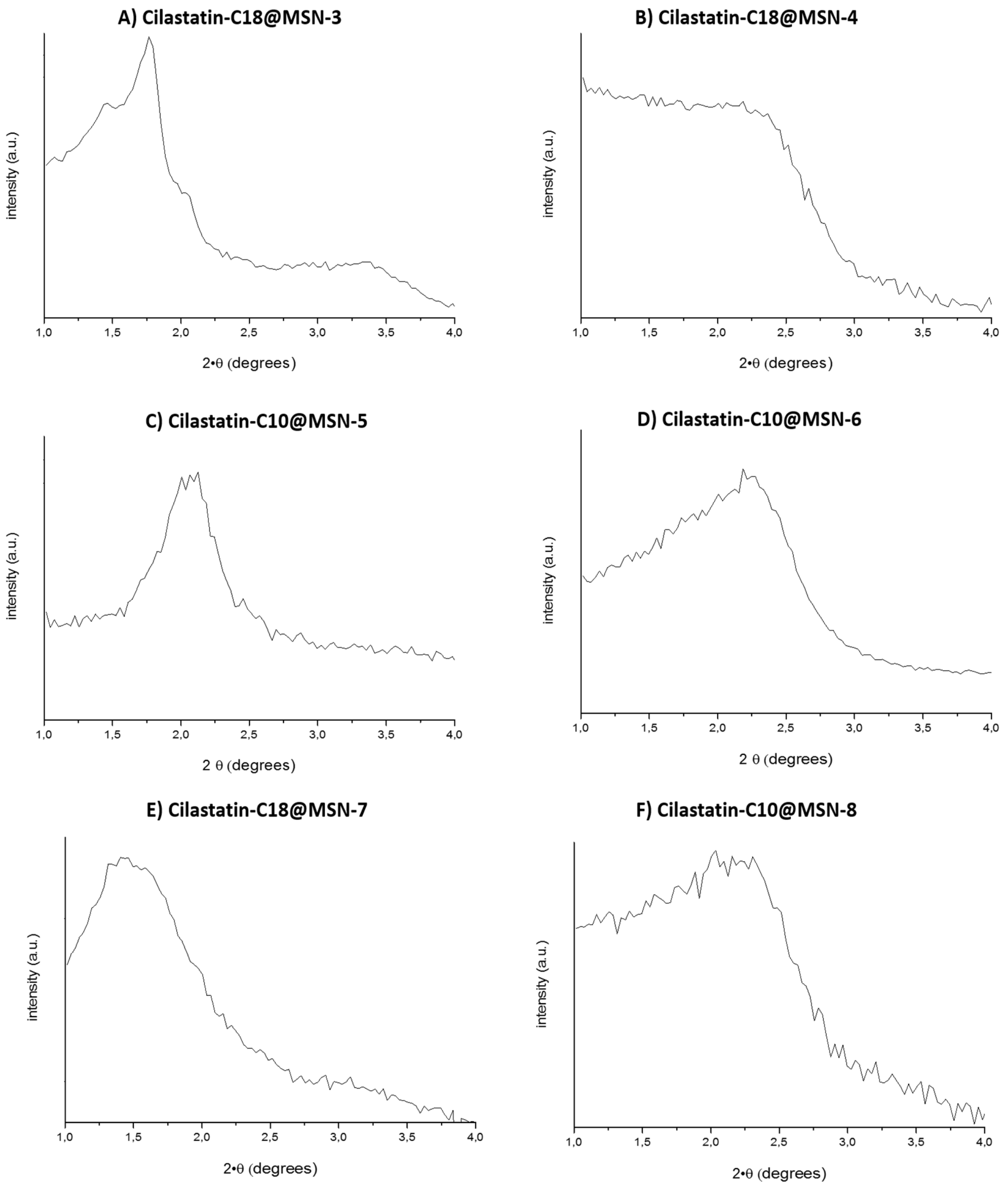

Figure S7. Low-angle X-ray diffraction patterns of cilastatin@MSNs. A) cilastatin-C18@MSN-3, B) cilastatin-C18@MSN-4, C) cilastatin-C10@MSN-5, D) cilastatin-C10@MSN-6, E) cilastatin-C18@MSN-7, F) cilastatin-C10@MSN-8.

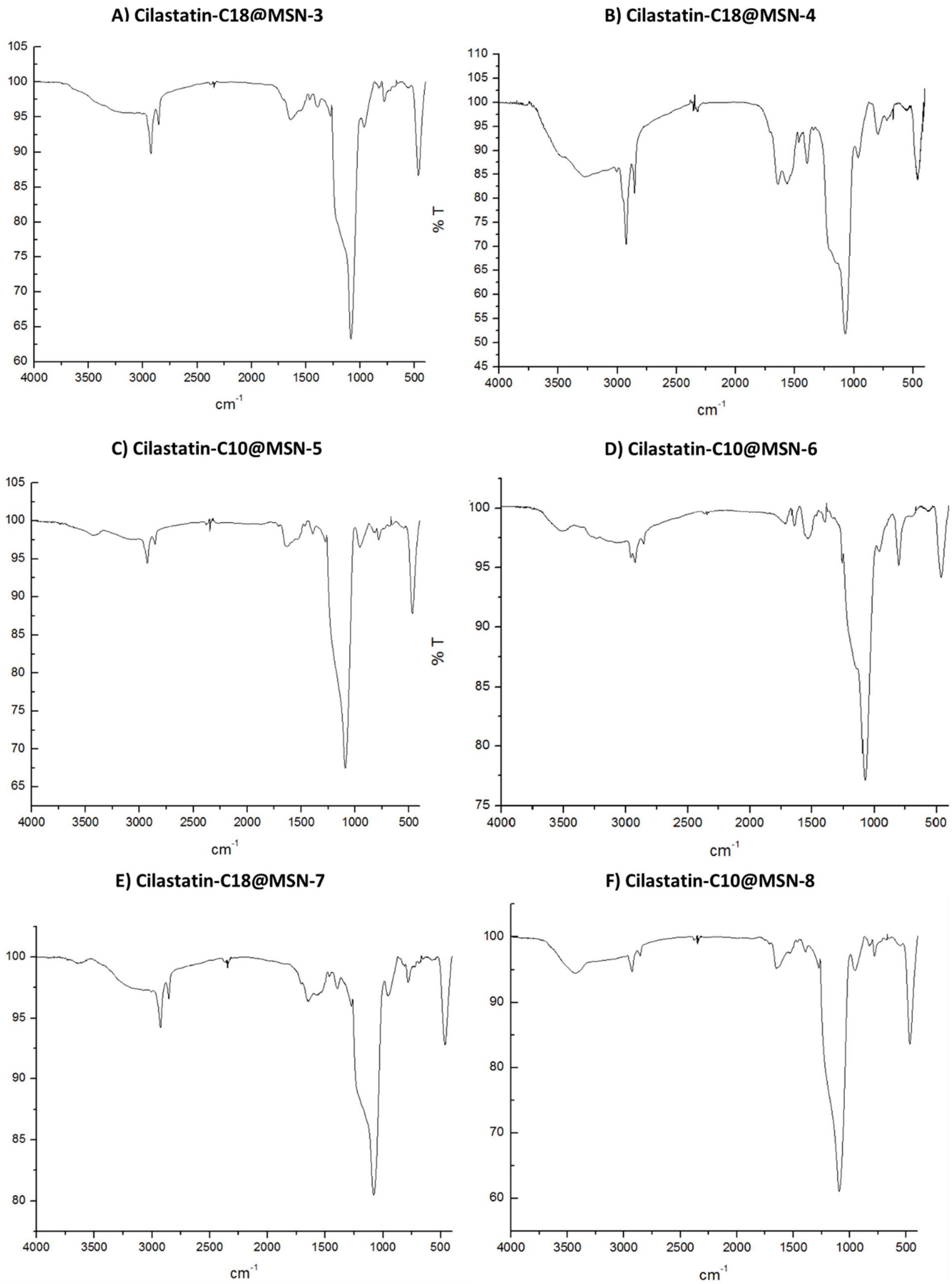

Figure S8. FTIR measurements of cilastatin@MSNs. A) cilastatin-C18@MSN-3, B) cilastatin-C18@MSN-4, C) cilastatin-C10@MSN-5, D) cilastatin-C10@MSN-6, E) cilastatin-C18@MSN-7, F) cilastatin-C10@MSN-8.

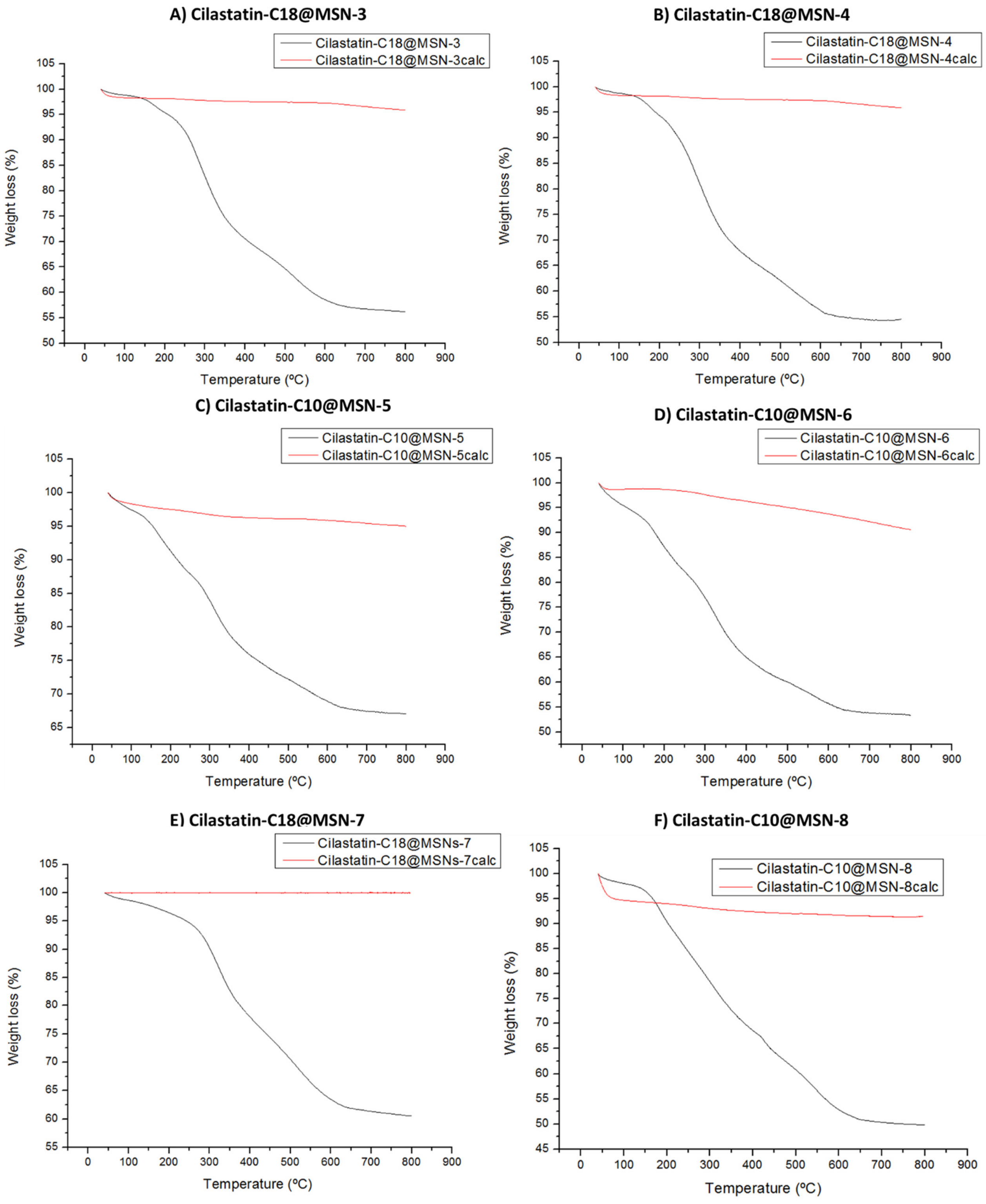

Figure S9. Thermogravimetric analysis of cilastatin@MSNs. A) cilastatin-C18@MSN-3, B) cilastatin-C18@MSN-4, C) cilastatin-C10@MSN-5, D) cilastatin-C10@MSN-6, E) cilastatin-C18@MSN-7, F) cilastatin-C10@MSN-8.

Table S1. Fitting parameters obtained by mathematical fitting of the cumulative release profiles of DSDA from cilastatin-C18@MSN-3 and cilastatin-C10@MSN-8 using Higuchi and Korsmeyer-Peppas drug release models.

**Material**	**Korsmeyer-Peppas Model** $f(t) = k_{KP} \cdot t^n$			**Higuchi Model** $f(t) = k_H \cdot t^{1/2}$	
	K_{KP}	n	R^2	K_H	R^2
Cilastatin-C18@MSN-3	2.35 ± 0.6	0.75 ± 0.04	0.98	7.12 ± 0.48	0.96
Cilastatin-C10@MSN-8	41.27 ± 0.6	0.14 ± 0.04	0.86	2.84 ± 1.00	0.61
